# Novel energy efficient RND inverter using quantum dot cellular automata in nanotechnology

**DOI:** 10.1038/s41598-023-49700-2

**Published:** 2024-01-02

**Authors:** Madhavi Repe, Sanjay Koli

**Affiliations:** 1 Department of Electronics and Telecommunication Engineering, G H Raisoni College of Engineering and Management, Wagholi, Pune, India; 2 Department of Electronics and Telecommunication Engineering, Dr. D. Y. Patil Unitech Society’s, Dr. D. Y. Patil Institute of Technology, Pimpri, Pune, India; 3 Department of Electronics and Telecommunication Engineering, Ajeenkya D. Y. Patil School of Engineering, Lohegaon, Pune, India

**Keywords:** Engineering, Electrical and electronic engineering

## Abstract

Quantum-Dot Cellular Automata (QCA) is a promising technology for designing high-performance and efficient logic circuits, surpassing traditional Complementary Metal Oxide Semiconductor approaches. In today’s digital era, the demand for digital circuits with high speed, device density, and energy efficiency is paramount. This paper focuses on the innovative Rotated Normal Cells with Displacement (RND) inverter model, employing normal and rotated cells with a 10 nm displacement through a cell interactive method. Digital circuits designed using the RND inverter exhibit superior performance compared to existing designs. The proposed RND inverter gate utilizes only four QCA cells, occupying a total area of 4525.55 nm^2^. With a total energy dissipation of 0.508 meV and an average energy dissipation per cycle of 0.0462 meV, it achieves a polarization of 9.77. The novel RND inverter demonstrates a 44% improvement in cell area and a 63% reduction in total area compared to current designs, offering enhanced energy efficiency with 0.26 improved polarization. The RND inverter and the digital circuits facilitate finding applications in efficiently constructing various components within Quantum Computers. Beyond quantum computing, the RND inverter proves applicable in designing Nano-sized electronic gadgets and temperature-controlled circuits, showcasing its versatility across diverse technological applications.

## Introduction

Quantum-dot Cellular Automata (QCA) stands out as a premier nanotechnology device, surpassing other Nanodevices in addressing the limitations of CMOS technology^1^. Renowned for its exceptional attributes, including high efficiency, elevated device density, and minimal power consumption, QCA operates within the Terahertz (THz) range. This technology adeptly represents binary information on its cells.

### Cells in QCA

Quantum dots in QCA are nanometer-scaled devices in the range of up to 10 nm. Quantum dots contain tiny droplets of free electrons. Quantum-dot cellular automata have two main concepts: quantum dot and cellular automata. Cellular automata is a term that has a grid of cells. The cells consist of four quantum dots at the four corners of it. The cells have a square shape with a standard size of 18 nm × 18 nm. The rectangular box around the cells differentiates them from the others. Cells can have an arrangement in an array form.

Cells have a finite number of states at a discrete time. The previous state of a cell and its immediate adjacent cells decide the state of the cell. The radius of effect in QCA is vital in selecting the number of adjacent cells. It helps to calculate the energy state of the cell and kink energy.

**Figure 1:**
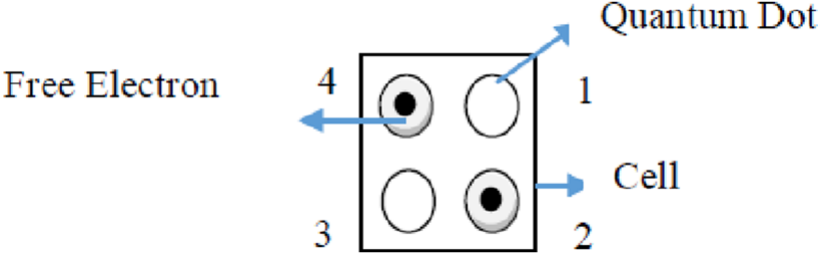
Structure of cell.

**Figure 2:**
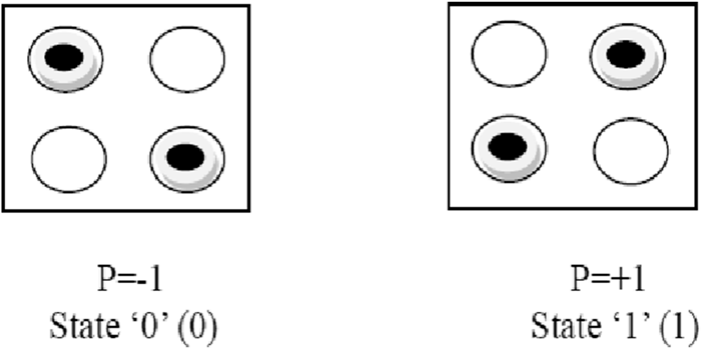
States of a cell.

The charging of a cell with two electrons makes these electrons take opposite positions. It is possible due to the effect of the columbic repulsion. There are only two opposite position possibilities of these electrons called cell polarization. These two states are nothing but binary 0 and binary 1. Figure 1 shows the structure of a cell. Figure 2 shows the states of a cell or cell polarization.

1 $${\text{P}}=\frac{\left({\text{P}}1+{\text{P}}3\right)-({\text{P}}2+{\text{P}}4)}{{\text{P}}1+{\text{P}}2+{\text{P}}3+{\text{P}}4}$$

Equation (1) states cell polarization and is denoted by ‘P’. It considers the presence or absence of an electron in a quantum dot. The quantum dots numbering is shown in Fig. 1.

### Cell-to-cell response function

**Figure 3:**
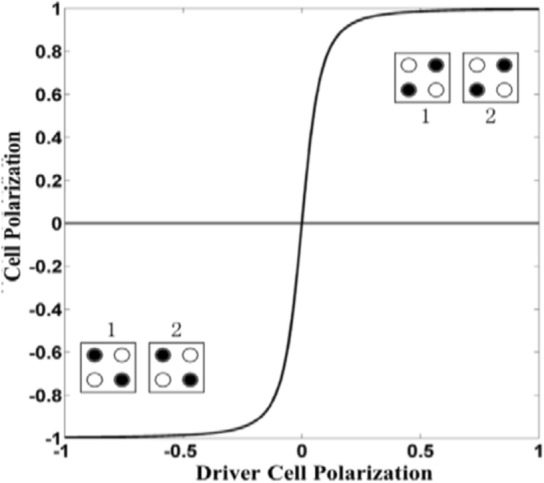
Cell to Cell response function^2^.

The communication between neighboring cells occurs via cell polarization, depicted in Fig. 3, where the cell-to-cell response function between two adjacent cells is nonlinear. The calculation of the cell-to-cell response is governed by the Schrödinger equation. In the scenario where cell number 2 exhibits a polarization of − 1, cell number 1 adopts a corresponding ground state configuration of − 1. Likewise, if cell number 2 displays a polarization of + 1, cell number 1 aligns itself with a ground state configuration of + 1^2^.

**Figure 4:**
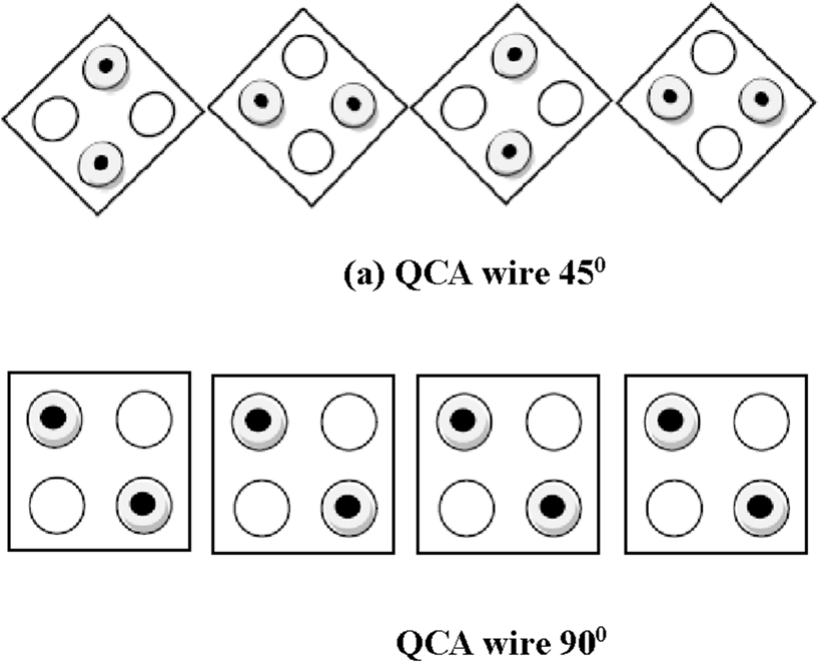
(**a**) QCA wire 45°. (**b**) QCA wire 90°.

Through the quantum tunnelling mechanism, electrons seamlessly traverse from one pair of quantum dots to another, prompting cells to dynamically switch between polarization states. This dynamic reflects the direct influence of neighboring cells’ polarizations on each other, fostering synchronization among adjacent cells. Consequently, an array of these synchronized cells functions as a conduit, akin to a wire, as illustrated in Fig. 4a,b, termed the QCA wire. In this QCA wire, information is transmitted from the driver cell to the subsequent cell.

Two distinct types of QCA wires exist, namely 45° and 90° wire, which are also known as rotated cells and normal cells, respectively, based on the arrangement of quantum dots within a cell. The subsequent sections of this work are outlined as follows: Section “QCA inverters” delves into existing inverter designs and provides comparisons; Section “Proposed RND (rotated normal cells with displacement) inverter model” illustrates the proposed QCA inverter layout along with its simulation result, mathematical proof, and simulated power dissipation. Section “Circuit implementation with proposed RND inverter” then outlines the circuit implementation with an innovative inverter and presents simulation results. Section “Result and discussion” discusses the results, and Section “Conclusion” serves as the conclusion.

## QCA inverters

The fundamental logic elements in QCA consist of the inverter and the majority voter gate. The inverter is characterized by a single input and a single output, where the output represents the inversion or negation of the input signal. Precisely, when provided with a binary input of 1, the output reflects 0, and conversely, for a binary input of 0, the output gives 1.

Numerous researchers have explored various methodologies for implementing the NOT gate or inverter, and several of these implementations are highlighted here.

In reference^3^, Tougaw et al. introduced a robust inverter model considered superior among various inverters. The Tougaw model is commonly called the fundamental inverter. Distinguished by its geometric symmetry, this inverter layout effectively bifurcates the signal into two paths, enabling improved polarization at the output.

The inverter model proposed by Lent et al. is characterized by reduced complexity, requiring less area and minimal polarization. In this model, inversion is achieved through cell displacement^4^. Frankish et al. also present an inverter model resembling Lent's, implementing it by strategically removing specific cells from the fundamental inverter through calculated adjustments^5^.

In the inverter model introduced by Navi et al., the authors have devised a configuration with two and three layers, deviating from the conventional single-layer inverter design^6^.

The authors in^7^ have introduced a less faulty and highly polarized inverter design. They utilized five rotated cells in crafting this innovative inverter. As per their findings, employing fewer cells reduces area and polarization. Additionally, the authors noted that increasing polarization can be achieved by incorporating more cells into the circuit, albeit this comes with a subsequent increase in kink energy between two QCA cells. Their work showcases inverter designs involving three, four, and five cells.

In reference^8^, the authors have introduced two innovative inverter designs. The first design utilizes three mixed cells, with normal cells as input and output, while the middle cell is rotating. This configuration is characterized by high polarization, efficiency, and a compact footprint. The second design, also featuring four cells, follows the same concept. In this variation, normal cells are positioned as input and output, while the middle two cells are rotating. The second design is robust and exhibits high polarization.

The AOI (AND OR Inverter) inverter model is a universal gate consisting of seven cells. It incorporates five input cells, one output cell, and one device cell. On the other hand, an MV5 gate is a five-input majority voter gate with additional inputs A and C compared to MV3. Its output is inverted, and it exhibits variation in polarization. However, due to marginal cell misplacement, its functionality can change^9^.

The NNI (NAND-NOR-INVERT) model comprises five cells, excluding the device cell^10^. It is also a universal gate. The NNI inverter model is more reliable than the universal AOI gate. In this layout, inputs A and B maintain a fixed polarization of “+ 1”.

The FNZ inverter model comprises 8 cells with three inputs and one output. Device density is achievable by translating 10 nm both vertically and horizontally for two input cells. As demonstrated by the authors, this model outperforms AOI and NNI Universal Gates in all parameters^5^. Mersede Zahmatkesh et al.^11^ employed only three cells in their model, where the middle cell is rotated 45° and has a displacement of 6 nm. This design occupies a small area.

A fault-tolerant inverter design employing a 2 × 2 tile structure is utilized in this context. This design optimizes the structural complexity of the inverter logic by incorporating four rotated cells within the 2 × 2 tile structure alongside input and output cells. During fabrication, the complexity of rotated cells is equivalent to that of normal cells. In coplanar wire crossing, normal and rotated cells do not interfere with each other^12^.

True to its name, the crossbar architecture inverter is employed explicitly in designing QCA circuits within a crossbar architecture. QCA cells are arranged like a majority voter gate^13^. The majority gate (MG) comprises nine cells, yet only four cells are utilized for inversion. The green-colored cells (four cells) are subjected to one clock, while a different clock triggers the blue-colored cells (five cells) to prevent overlap and achieve inversion. The two wires should be diagonal, providing high reliability and stability.

A comprehensive comparison of all existing inverters is presented in Table 1.

**Table 1:** Comparison of all existing inverters.

Inverter model	Cell count	Total area in nm^2^	Polarization achieved	Energy dissipation in meV (Total energy, average energy)
Tougaw et al.^3^ Inverter Model	8	5684	0.775	3.02, 0.275
Lent et al.^4^ Inverter Model	4	2964	0.559	2.17, 0.197
Farazkish et al.^5^ Inverter Model	10	7298	0.954	3.08, 0.280
Navi et al.^6^ Inverter two layer design Model	6	1764	0588	Multilayer crossover
Navi et al.^6^ Inverter Three layer design Model	8	1764	0.842	Multilayer crossover
Angshuman et al.^7^ Inverter model	4	5576	0.994	0.776, 0.0705
Khanday et al.^8^ Inverter Model with 3 cells	3	1624	0.879	1.07, 0.0973
Khanday et al.^8^ Inverter Model with 4 cells	4	2204	0.969	1.10, 0.097
AOI (And-Or-Inverter) Inverter Model^9^	7	12,744	+ 0.525 and − 0.629	3.64, 0.0331
NNI (NAND-NOR-INVERT) Model^10^	5	4524	+ 0.948 to − 0.951	1.00, 0.0912
FNZ Inverter Model^5^	8	6084	0.931	0.861, 0.0782
Inverter by Mersede Zahmatkesh et al.^11^	3	2378	0.879	1.07, 0.0973
Fault Tolerant Inverter design^12^	4	4384	0.969	1.83, 0.167

Table 1 provides a comprehensive comparison of all existing inverter implementations conducted by researchers up to the present. Various methods are available for inverter implementation, each aiming to achieve optimization. Every design implementation employs its approach to placing cells in crossings, such as coplanar and multilayer crossover. Therefore, this paper emphasizes a novel and optimized RND inverter model and digital circuits designed to minimize energy dissipation and enhance device density, speed, and polarization.

## Proposed RND (rotated normal cells with displacement) inverter model

**Figure 5:**
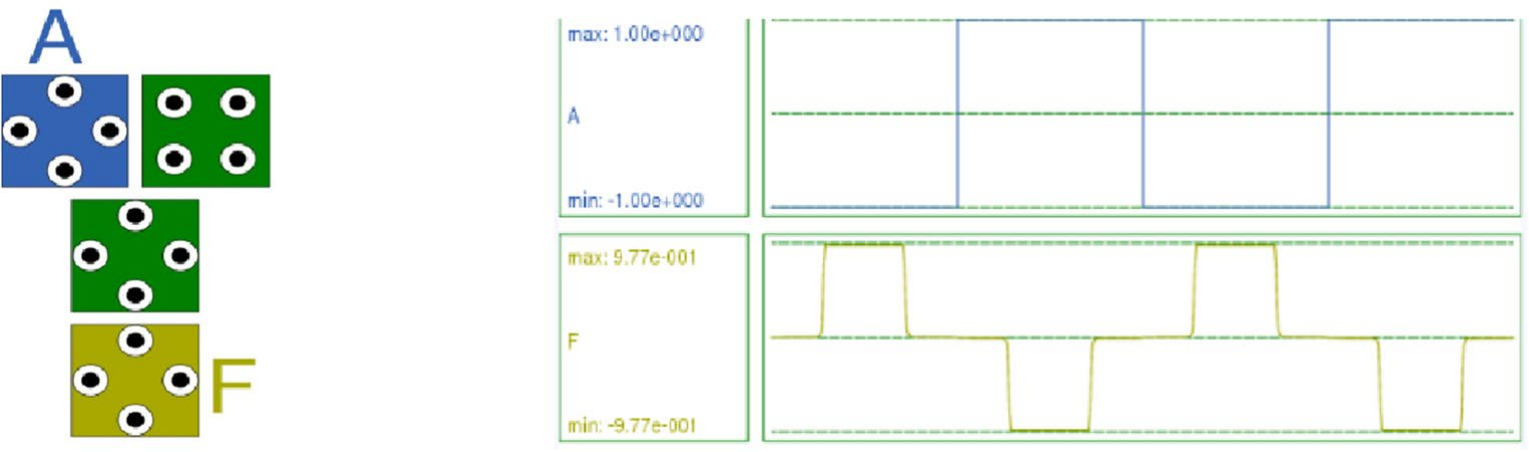
QCA Layout and Simulation Result of the proposed inverter.

The proposed RND inverter comprises four cells and is implemented using a combination of normal and rotated cells. This inverter exhibits a smaller area, fewer cells, and lower energy dissipation than the standard NOT gate. The cells are horizontally displaced by 10 nm, and this arrangement and displacement yield optimized simulated results in QCADesigner 2.0.3. The QCA layout and simulation results are depicted in Fig. 5. QCADesigner 2.0.3 employs numerical methods to simulate the quantum behaviour of electrons within quantum dots and their interactions, featuring both a Bistable simulation Engine and a Coherence vector simulation Engine. Quantum simulation tools like Qiskit can be adapted to model QCA circuits. The QCADesigner-E software tool, an extended version of QCADesigner, obtains the total and average energy dissipation per cycle for the QCA layout.

### Mathematical analysis

**Figure 6:**
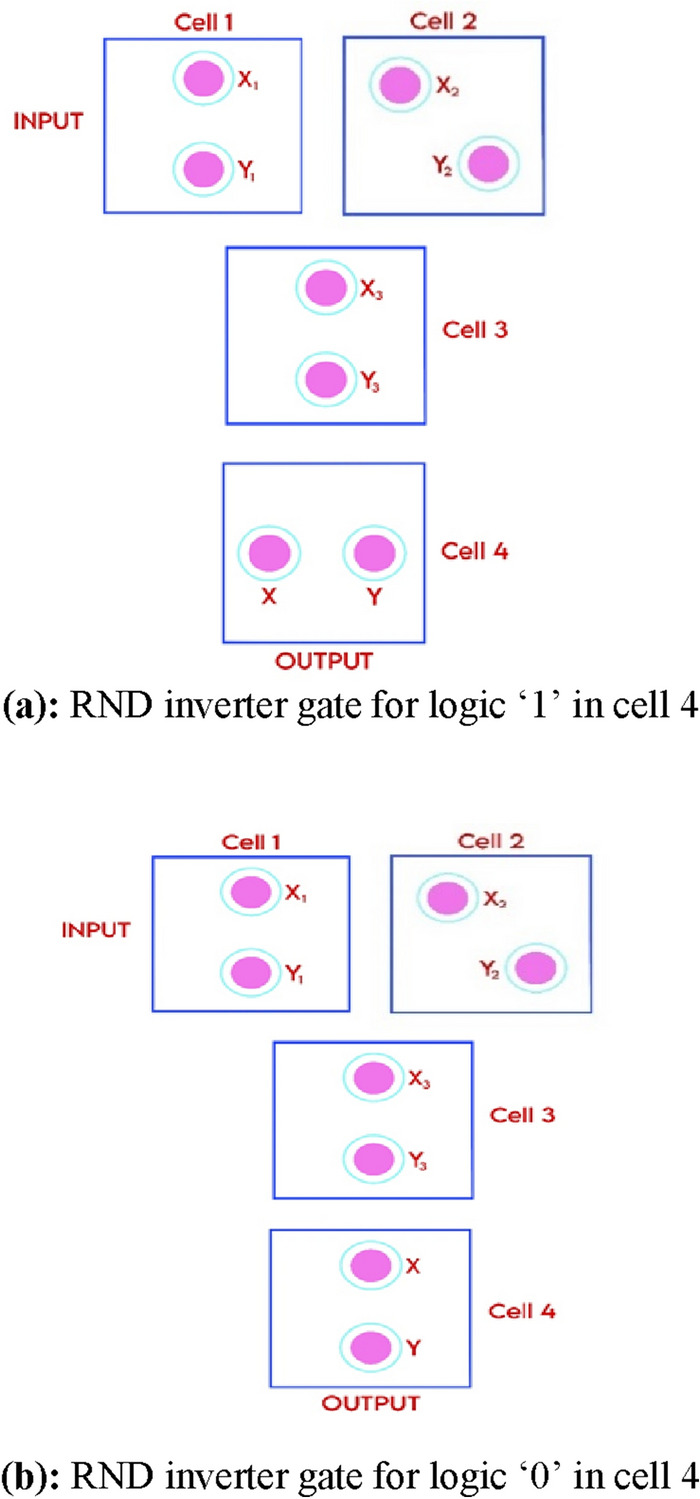
(**a**) RND inverter gate for logic ‘1’ in cell 4. (**b**) RND inverter gate for logic ‘0’ in cell 4.

The mathematical analysis of the energy dissipation of the RND inverter gate is presented in this study. The provided proof demonstrates that the output is an inversion of the input when using this RND inverter gate. This inversion arises from the arrangement of cells designed to enhance stability and minimize potential energy. The cell dimensions are assumed to be 18 × 18 nm, with a 2 nm separation between neighboring cells. Figure 6a,b illustrate the square shape representing the QCA cell, with filled circles indicating the positions of electrons within that cell.

Equation (2) is employed to calculate the potential energy between two electron charges. In this equation, U represents the potential energy, k is the Boltzmann constant (9 × 10^9^), Q1 and Q2 denote the charges of electrons (1.602 × 10^−19^), and ‘r’ signifies the distance between the two electric charges^14,15^.

2 $$U=k \frac{Q1.Q2}{r}$$

By substituting the values of Q1, Q2, and k into Eq. (2), the potential energy is expressed in simplified form in Eq. (3).

3 $$U = ~\frac{{23.04\; \times \;10^{{ - 29}} }}{r}$$

The potential energy between two cells is computed by rounding up the dots in each cell as indicated by Eq. (4).

4 $$UT=\sum_{i=1}^{n}Ui$$

If a logic ‘0’ is applied at the input side to cell one, then cell two and cell three will follow the logic as indicated. Let us determine the position of electrons in the output cell. The potential energy at cell four is calculated for both state (a) and state (b), as illustrated in Fig. 6a,b, respectively. The state that yields the minimum potential energy is considered the most stable.

As depicted in Fig. 6a, the potential energy of electron ‘X’ of cell 4 for X_1_, X_2_, X_3_, and Y_1_, Y_2_, and Y_3_ of cells 1, 2, and 3 is computed. Similarly, the potential energy of electron ‘Y’ of cell 4 for X_1_, X_2_, X_3_, and Y_1_, Y_2_, and Y_3_ of cells 1, 2, and 3 is calculated. The total potential energy of electron 'X' for all other cells is then computed using Eq. (4) to obtain ${U}_{{T}^{11}}$, and the potential energy of electron ‘Y’ for all other cells is added using Eq. (4) to get ${U}_{{T}^{12}}$. The total energy UT1 for the Fig. 6a representation is the sum of ${U}_{{T}^{11}}$ and ${U}_{{T}^{12}}$. These calculations are detailed in assumption 1. Similarly, assumption 2 illustrates the total energy UT1 for the Fig. 6b representation.

#### Assumption 1

If cell-4 is logic ‘1’ as shown in Fig. 6a

Figure 6a (Electron x)

$$U{1 = }\frac{A}{{r_{1} }} = \frac{{23.04\; \times \;10^{ - 29} }}{{44.570\; \times \;10^{ - 9} }} = 0.516\; \times \;10^{ - 20 } J$$

$$U{2 = }\frac{A}{{r_{2} }} = \frac{{23.04\; \times \;10^{ - 29} }}{{46.360\; \times \;10^{ - 9} }} = 0.496\; \times \;10^{ - 20 } J$$

$$U{3 = }\frac{A}{{r_{3} }} = \frac{{23.04\; \times \;10^{ - 29} }}{{25.347\; \times \;10^{ - 9} }} = 0.908\; \times \;10^{ - 20 } J$$

Figure 6a (Electron y)

$$U{1 = }\frac{A}{{r_{1} }} = \frac{{23.04\; \times \;10^{ - 29} }}{{36.912\; \times \;10^{ - 9} }} = 0.624\; \times \;10^{ - 20 } J$$

$$U{2 = }\frac{A}{{r_{2} }} = \frac{{23.04\; \times \;10^{ - 29} }}{{36.390\; \times \;10^{ - 9} }} = 0.633\; \times \;10^{ - 20 } J$$

$$U{3 = }\frac{A}{{r_{3} }} = \frac{{23.04\; \times \;10^{ - 29} }}{{14.983\; \times \;10^{ - 9} }} = 1.531\; \times \;10^{ - 20 } J$$

$$U_{{T^{11} }} = \mathop \sum \limits_{i = 1}^{6} U{\text{i}} = 1.92\; \times \;10^{{ - 20{ }}} J$$

$${\text{U}}_{{{\text{T1}}}} {\text{ = 4}}.714\; \times \;10^{{ - 20}} {\text{J}}$$

#### Assumption 2

If cell-4 is logic ‘0’ as shown in Fig. 6b

Figure 6b (Electron x)

$$U{1 = }\frac{A}{{r_{1} }} = \frac{{23.04\; \times \;10^{ - 29} }}{{39.051\; \times \;10^{ - 9} }} = 0.589\; \times \;10^{ - 20 } J$$

$$U{2 = }\frac{A}{{r_{2} }} = \frac{{23.04\; \times \;10^{ - 29} }}{{28.327\; \times \;10^{ - 9} }} = 0.813\; \times \;10^{ - 20 } J$$

$$U{3 = }\frac{A}{{r_{3} }} = \frac{{23.04\; \times \;10^{ - 29} }}{{20\; \times \;10^{ - 9} }} = 1.152\; \times \;10^{ - 20 } J$$

$$U_{{T^{11} }} = \mathop \sum \limits_{i = 1}^{6} U{\text{i}} = 2.554\; \times \;10^{{ - 20{ }}} J$$

$$U_{{T^{12} }} = \mathop \sum \limits_{i = 1}^{6} U{\text{i}} = 2.794\; \times \;10^{{ - 20{ }}} J$$

Figure 6b (Electron y)

$$U{1 = }\frac{A}{{r_{4} }} = \frac{{23.04\; \times \;10^{ - 29} }}{{41\; \times \;10^{ - 9} }} = 0.561\; \times \;10^{ - 20 } J$$

$$U{2 = }\frac{A}{{r_{5} }} = \frac{{23.04\; \times \;10^{ - 29} }}{{34.432\; \times \;10^{ - 9} }} = 0.669\; \times \;10^{ - 20 } J$$

$$U{3 = }\frac{A}{{r_{6} }} = \frac{{23.04\; \times \;10^{ - 29} }}{{20\; \times \;10^{ - 9} }} = 1.152\; \times \;10^{ - 20 } J$$

$$U_{{T^{12} }} = \mathop \sum \limits_{i = 1}^{6} U{\text{i}} = 2.382\; \times \;10^{{ - 20{ }}} J$$

$$U_{{T1}} = 4.936\; \times \;10^{{ - 20}} J U_{{T1}} = 4.936\; \times \;10^{{ - 20}} J$$

The analysis reveals that the potential energy of cell four in Fig. 6a is lower than in Fig. 6b. Therefore, cell four is considered at logic ‘1’, indicating an input inversion at the output. Similarly, if cell 1 is at logic ‘1’, cell four will yield logic ‘0’.

### Power dissipation and polarization

**Figure 7:**
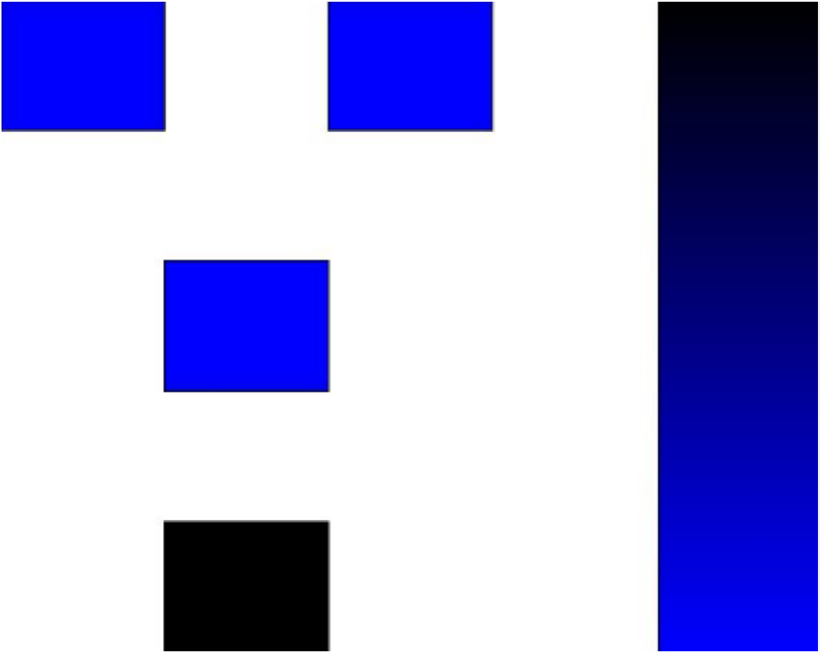
Polarization analysis for input ‘1’ at 0.5 meV, 1.0 meV and 1.5 meV.

For further insights into the power dissipation and polarization of the proposed RND inverter, the QCAPro software provides a detailed power dissipation analysis at various kink energy levels. The images depicting power and polarization levels can be observed within the QCAPro software. Figure 7 displays the polarization analysis at 0.5 meV, 1.0 meV, and 1.5 meV levels. These values are obtained at a temperature (T) of 2 K.

**Figure 8:**
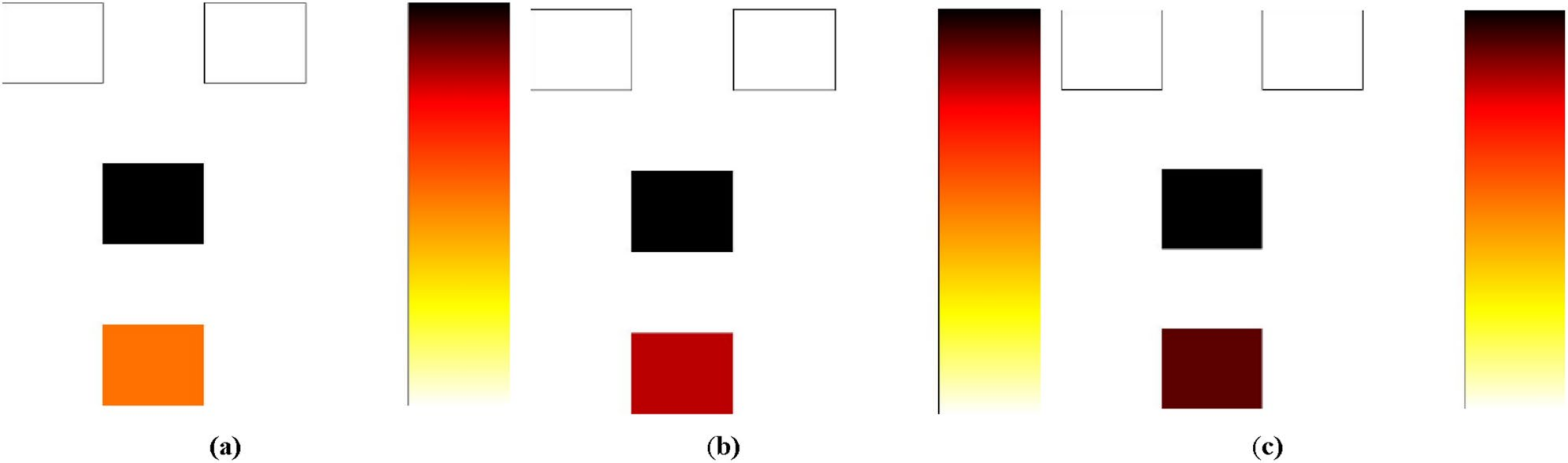
(**a**) Power dissipation analysis at 0.5 meV (**b**) Power dissipation analysis at 1 meV (**c**) Power dissipation analysis at 1.5 meV.

Figure 8 illustrates the power dissipation analysis at 0.5 meV, 1.0 meV, and 1.5 meV levels for the proposed RND inverter.

The proposed design accurately reflects each input combination’s polarization at different kink energy levels, as depicted in Fig. 7. Binary values are represented using only two colors: the black color cell denotes logic ‘0’, while the blue color cell represents logic ‘1’.

Figure 8a–c depict the power dissipation analysis for the proposed RND inverter. The Orange-colored cell represents the lowest level of energy dissipation, while the Black color indicates the highest level of energy dissipation by the cell^16^. Figure 8a–c reveal that only a single Black cell is subject to displacement, and the remaining cells dissipate minimal energy.

## Circuit implementation with proposed RND inverter

The circuit implementations using the proposed RND inverter for designing AND, OR, NOR, NAND, XOR gates, 2:1 Multiplexer, Half adder, and Half subtractor are highlighted in this section. Basic gates, universal gates, XOR gates, multiplexer blocks, and adder/subtractors are essential for constructing circuits in digital Nanoelectronics. Optimizing these fundamental blocks contributes to the miniaturization of complex circuits. This paper presents all gates, including the XOR gate, 2:1 multiplexer, Half adder, and Half subtractor, using the RND inverter, cell interaction methodology, and modifications in efficient existing layouts to optimize energy dissipation. The circuits avoid multilayer crossovers, exhibit low energy dissipation, and demonstrate superior polarization compared to previous implementations.

### Basic gates

**Figure 9:**
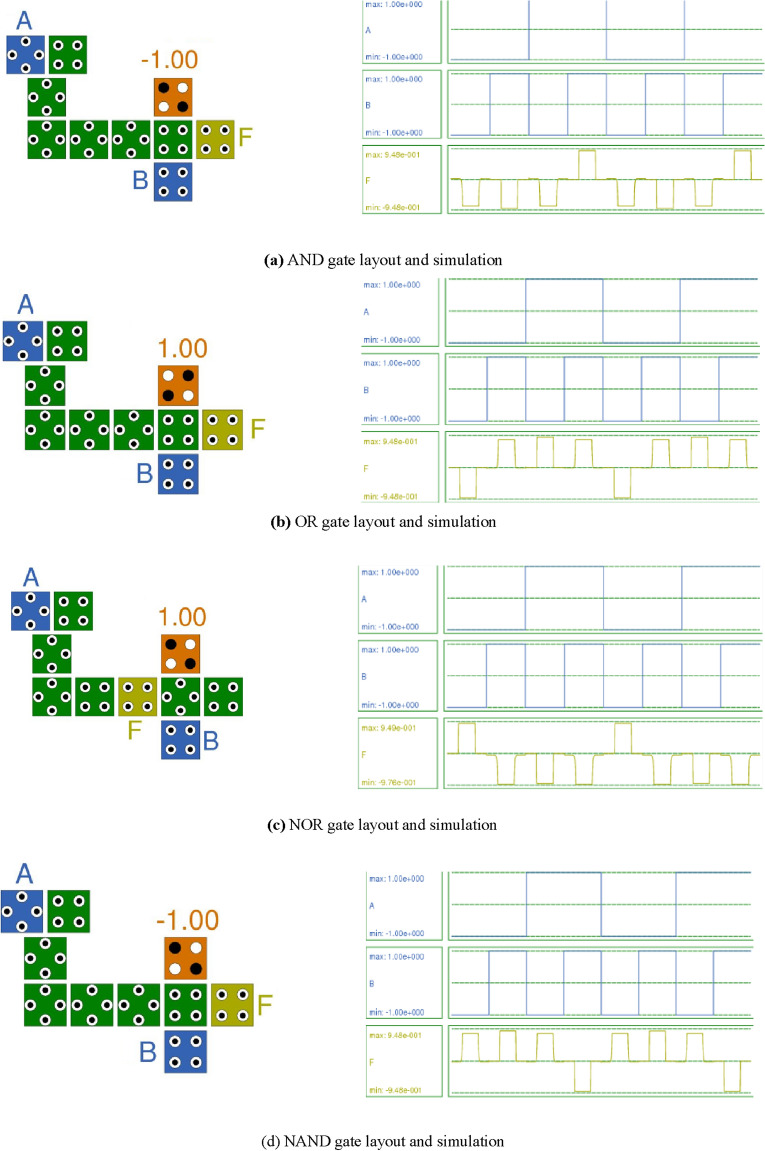
(**a**) AND gate layout and simulation. (**b**) OR gate layout and simulation. (**c**) NOR gate layout and simulation. (**d**) NAND gate layout and simulation.

In QCA, the design layout of the standard AND gate, OR gate, NOR gate, and NAND gate utilizes the majority voter gate. An optimal design of AND, OR, NOR, and NAND gates is achieved by properly arranging normal and rotated cells along with the RND inverter. Figure 9a–d depict all these gates' layout and simulation results.

The simulation results presented in Fig. 9a–d validate the truth table of the respective gate. For instance, in the case of the NAND gate shown in Fig. 9d, for two input binary values ‘A’ and ‘B’, the output ‘F’ indicates that if either input is binary ‘0’, then the output is high, thereby confirming the truth table of the NAND gate.

Table 2 provides a comparison between the proposed RND inverter and the standard inverter in the Section “Results and Discussion”. It also compares the AND, OR, NAND, and NOR gates based on the number of iterations required to obtain the simulation result, total and average energy dissipation per cycle, and simulation time. The table clearly indicates that the proposed RND inverter and the other gates designed using it outperform in terms of all the mentioned parameters.

**Table 2:** Comparison of standard gates with proposed RND inverter gate.

Gate	No. of Iterations	Total energy dissipation (meV)	Average energy dissipation per cycle (meV)	Total simulation time (second)
Standard NOT Gate	6	3.32	0.302	8
Proposed RND inverter Gate	8	0.50	0.0462	3
Standard AND Gate	5	7.90	0.718	5
AND with the proposed RND inverter Gate	3	4.14	0.377	5
Standard OR Gate	6	8.30	0.755	5
OR with the proposed RND inverter Gate	4	4.87	0.442	5
Standard NAND Gate	7	9.01	0.819	9
NAND with proposed RND inverter Gate	6	2.72	0.247	6
Standard NOR Gate	13	9.28	0.844	9
NOR with proposed RND inverter Gate	3	1.64	0.149	5

For example, the NAND gate with the proposed RND inverter requires six iterations, has a total energy dissipation of 2.72 meV, an average energy dissipation of 0.247 meV, and a total simulation time of 6 s. It demonstrates improvements in all parameters compared to the standard NAND gate.

### XOR gate

**Figure 10:**
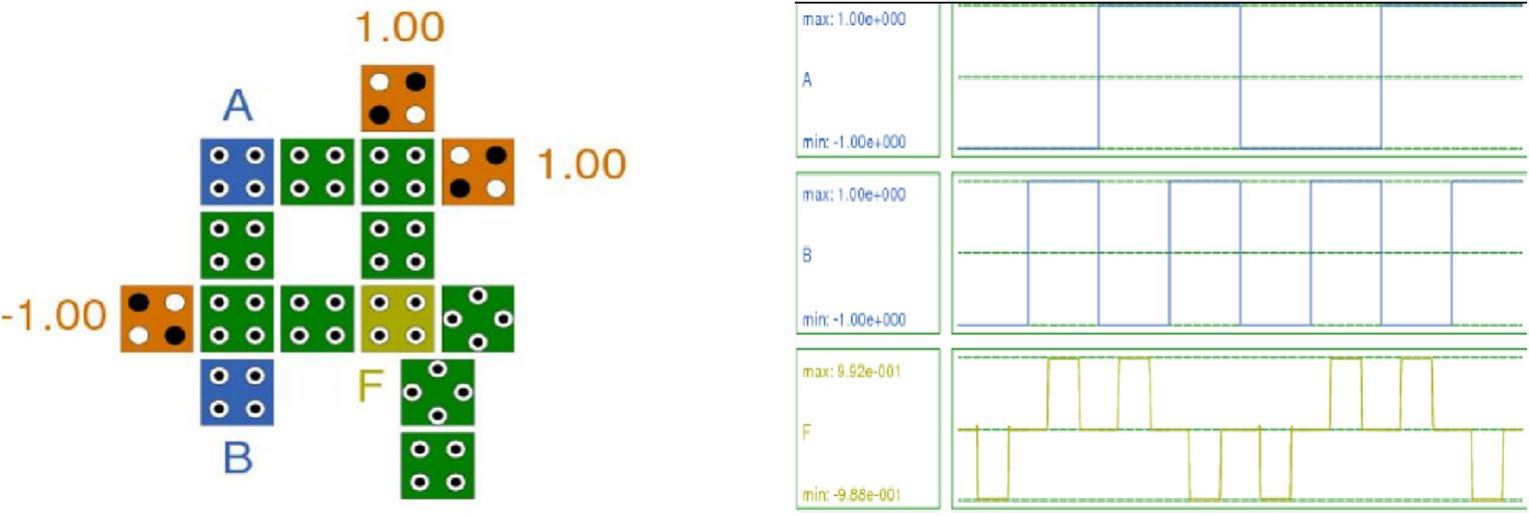
Layout and Simulation Result of XOR gate.

Figure 10 presents the QCA layout and simulation results of the XOR gate.

The existing XOR gate circuit^17^ comprises 14 cells, an area of 24,557.02 nm^2^, total energy dissipation of 11.7 meV (Error: +/− − 1.24 meV), and an average energy dissipation per cycle of 1.07 meV (Error: +/− − 0.113 meV). It exhibits a polarization of + 9.50 and − 9.50.

In comparison, the XOR gate with the proposed RND inverter utilizes 15 cells with a total area of 25,617.96 nm^2^. It necessitates a total energy dissipation of 11.3 meV (Error: +/− − 1.20 meV) and an average energy dissipation per cycle of 1.03 meV (Error: +/− − 0.109 meV). The achieved polarization is the best among all existing XOR gate implementations, with values of + 9.92 and − 9.89. These values indicate an improvement in both energy dissipation and polarization.

### 2:1 multiplexer

**Figure 11:**
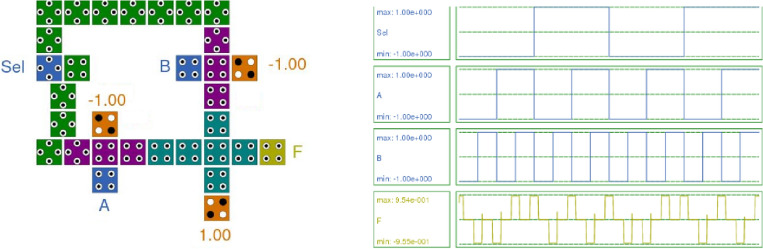
Layout and Simulation Result of 2:1 Multiplexer.

Figure 11 presents the QCA layout and simulation results of the 2:1 multiplexer. The simulation results indicate that when the two inputs are different, the output is set to one, and if both inputs are the same, the output is zero. Additionally, when the select line ‘Sel’ is zero, the output corresponds to input ‘A’, while with ‘Sel’ as one, input ‘B’ is selected at the output. The simulation results further reveal that the output is delayed by 0.75 clock cycles.

For the 2:1 Multiplexer QCA layout with the proposed RND inverter, the total energy dissipation is 11.1 meV (Error: +/− − 1.01 meV), and the average energy dissipation is 1.01 meV (Error: +/− − 0.0920 meV). It utilizes 31 cells with an area of 47,360 nm^2^. The polarization for output ‘F’ is + 9.54 and − 9.55, and the output has a latency of 0.75 clock cycles.

In contrast, the existing 2:1 multiplexer^18^ comprises 15 cells with an area of 20,178.59 nm^2^. The total energy dissipation is 15.9 meV (Error: +/− − 1.65 meV), and the average energy dissipation per cycle is 1.44 meV (Error: +/− − 0.150 meV). The polarization for output ‘F’ is + 9.47 and − 9.44, and the output has a latency of 0.5 clock cycles.

The achieved polarization is higher than the existing 2:1 multiplexer, which utilizes a rotated cell with displacement. The proposed 2:1 multiplexer demonstrates superiority in terms of energy dissipation and polarization.

### Half adder

**Figure 12:**
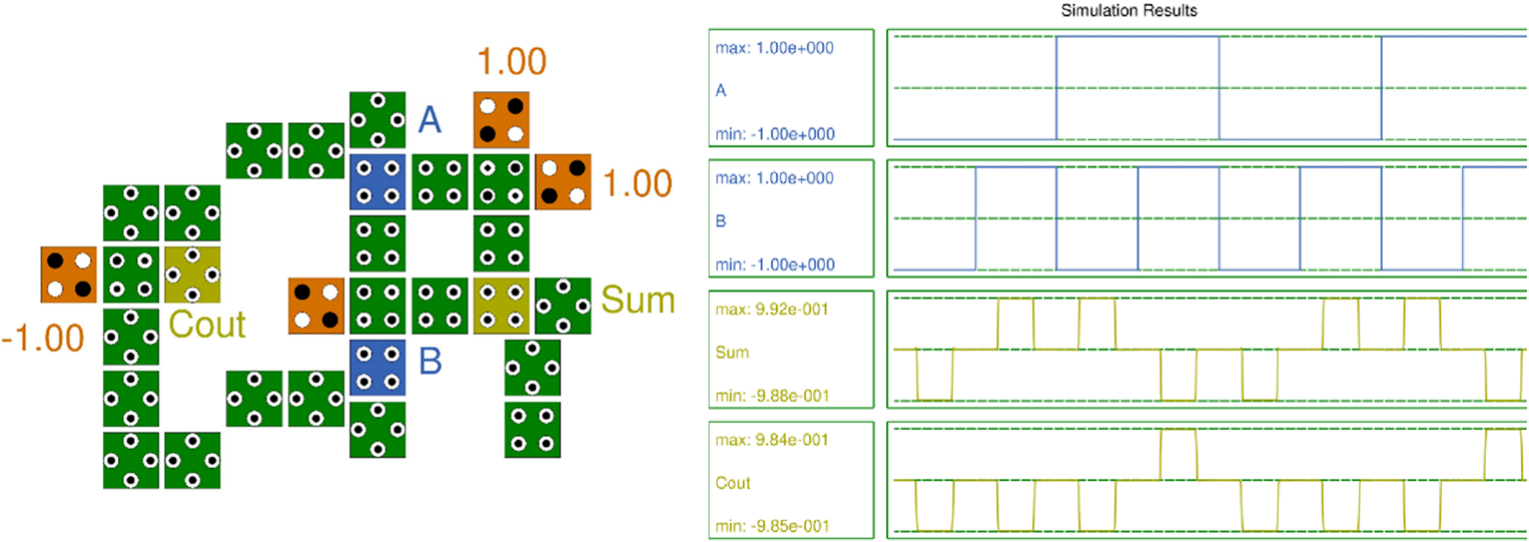
Layout and Simulation Result of Half Adder.

Figure 12 illustrates the QCA layout and simulation results of the Half adder. The half adder combines two binary bits, ‘A’ and ‘B’, producing two outputs: Summation ‘Sum’ and carry ‘Cout’. The simulation results validate the truth table of the half-adder.

For the half-adder QCA layout with the proposed RND inverter, the total energy dissipation is 15.6 meV (Error: +/− − 1.56 meV), and the average energy dissipation per cycle is 1.42 meV (Error: +/− − 0.142 meV). It utilizes 30 cells with an area of 40,589.84 nm^2^. The polarization for SUM is + 9.92 and − 9.88, and for COUT, it is + 9.84 and − 9.85. Here, both SUM and COUT have a latency of 0.25.

In comparison, the recently available half-adder circuit^19^ comprises 39 cells with an area of 50,348.74 nm^2^. The total energy dissipation is 17.6 meV (Error: +/− − 1.67 meV), and the average energy dissipation per cycle is 1.60 meV (Error: +/− − 0.152 meV). The polarization for SUM and COUT is the same, at + 9.54 and − 9.55. Here, both SUM and COUT have a latency of 0.75.

The achieved polarization is higher compared to all existing half-adders. The parametric values demonstrate that the half-adder QCA layout designed is superior to the existing half-adder in terms of energy dissipation, polarization, number of cells, area, and latency.

### Half subtractor

**Figure 13:**
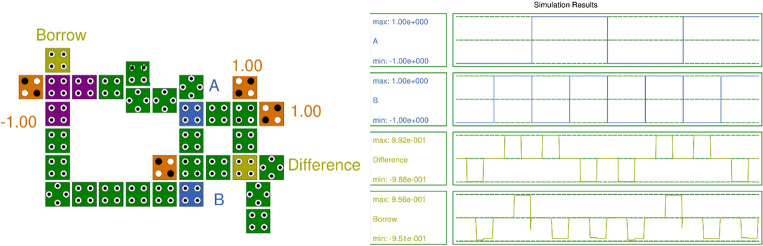
Layout and Simulation Result of Half Subtractor.

Figure 13 displays the QCA Layout and simulation results of the Half Subtractor. The half subtractor deducts two binary bits, ‘A’ and ‘B’, yielding two outputs: the subtraction as ‘Difference’ and the borrow ‘Borrow’. The simulation results validate the truth table of the half subtractor.

For the half-subtractor QCA layout with the proposed RND inverter, the total energy dissipation is 21.0 meV (Error: +/− − 2.17 meV), and the average energy dissipation per cycle is 1.91 meV (Error: +/− − 0.198 meV). It employs 32 cells with an area of 59,340.00 nm^2^. The polarization for ‘Difference’ is + 9.92 and − 9.89, and for ‘Borrow’, it is + 9.56 and − 9.38. Here, 'Difference' has a latency of 0.25, and ‘Borrow’ has a latency of 0.5.

In contrast, the half subtractor QCA layout in^20^ utilizes a multilayer crossover involving 55 cells, an area of 0.0504 µm^2^, and a latency of three clock phases or 0.75 clock cycles. This layout is complex and costly due to multilayer crossover, and the latency is also higher than that of the proposed half subtractor.

The half-subtractor QCA layout in^21^ features 38 cells with an area of 75,978.00 nm^2^. The total energy dissipation is 23.9 meV (Error: +/− − 2.38 meV), and the average energy dissipation per cycle is 2.18 meV (Error: +/− − 0.216 meV). The polarization for ‘Difference’ is + 9.53 and − 9.52, and for ‘Borrow’, it is + 9.52 and − 9.52. Here, ‘Difference’ has a latency of 0.5, and ‘Borrow’ has a latency of 0.75.

The achieved polarization is high compared to all existing half-subtractors. The parametric values indicate that the half-subtractor QCA layout designed is superior to existing half-subtractors in terms of energy dissipation, polarization, number of cells, area, and latency.

## Result and discussion

Table 2 provides detailed information on energy dissipation in the proposed RND inverter gate and other gates implemented using the RND inverter. The data in the table leads to the conclusion that both the proposed RND inverter gate and the other gates implemented using this RND inverter gate exhibit superior energy dissipation compared to all other existing gates.

Table 2 concludes that there is less energy dissipation in all the gates using the proposed RND gate. The energy dissipation in the proposed inverter is less than the inverter designed by Tougaw et al.^3^. The total energy dissipation is 0.508 meV with a minor error of +/− − 0.0352 meV. The average energy dissipation per cycle is 0.0462 meV with a minor error of +/− − 3.20 µeV. In comparison, the total and average energy dissipation per cycle of the QCA layout in^3^ are 3.02 meV with a minor error of +/− − 0.290 meV and 0.275 meV with a minor error of +/− − 0.0264 meV, respectively.

Table 3 provides the parametric analysis of AND, OR, NAND, and NOR gates implemented using the RND inverter gate.

**Table 3:** Parametric analysis of digital logic gates with proposed RND inverter gate.

Digital logic gate	Number of cells	Total area (nm^2^)	Polarization	Type of cells used
Proposed RND inverter Gate	4	4525.55	9.77	Mixed cells
AND Gate	10	15,243.58	9.48	Mixed cells
OR Gate	10	15,243.58	9.48	Mixed cells
NAND Gate	10	15,243.58	9.48	Mixed cells
NOR Gate	10	15,162	9.49, − 9.76	Mixed cells

The data in Table 3 concludes that the polarization achieved with the proposed RND inverter is the best.

Table 4 presents the parametric analysis of AND, OR, NAND, and NOR gates implemented using a standard NOT gate.

**Table 4:** Parametric analysis of digital logic gates with standard NOT gate.

Digital logic gate	Number of cells	Total area (nm^2^)	Polarization	Type of cells used
Standard NOT Gate^3^	9	7198	9.51	Normal cells
AND Gate^3^	9	19,941	9.54	MV3 Gate
OR Gate^3^	9	19,941	9.54	MV3 Gate
NAND Gate	16	22,221.02	9.51	MV3 and standard NOT Gate
NOR Gate	16	22,812.50	9.51	MV3 and standard NOT Gate

Table 5 illustrates the improvement in various parameters of digital logic gates achieved by the proposed RND inverter gate compared to gates implemented using a standard NOT gate.

**Table 5:** Improvement in the parametric analysis of digital logic gates.

Digital logic gate	Number of cells			Total area (nm^2^)			Energy dissipation (Total, average)		
	Standard	Proposed	Improvement (%)	Standard	Proposed	Improvement (%)	Standard (meV)	Proposed (meV)	Improvement (%)
Proposed RND inverter Gate	9	4	44	7198	4525.55	62.8	3.32, 0.302	0.508, 0.0462	15.30
									15.29
AND Gate	9	10	–	19,941	15,243.58	76.4	7.90, 0.718	4.14, 0.377	52.40
									52.50
OR Gate	9	10	–	19,941	15,243.58	76.4	8.30, 0.755	4.87, 0.442	58.67
									58.54
NAND Gate	16	10	62.5	22,221.02	15,243.58	68.5	9.01, 0.819	2.72, 0.247	30.18
									30.15
NOR Gate	16	10	62.5	22,812.50	15,162	66.4	9.28, 0.844	1.64, 0.149	17.67
									17.65

Table 5 concludes that the RND inverter yields the best results in terms of the number of cells, total area, and energy dissipation.

## Conclusion

The proposed novel RND inverter gate is the best among all implemented inverters. It demonstrates superior device density, polarization, total area, and energy consumption results. In today’s era, lower energy dissipation is a more demanding criterion for implementing digital circuitry. The use of this inverter model extends its benefits to other circuit designs, such as XOR gates, 2:1 multiplexers, half adders, and half subtractors, showcasing improvements in polarization and device density.

The novel RND inverter utilizes a displacement of 10 nm in constructing the design layout. During fabrication, proper care is required to place these cells with displacement and ensure connectivity with other parts of the circuits. It achieves a remarkable improvement of 44% in the count of the number of cells and a 62.5% improvement in building NAND and NOR gates compared to existing gates. Furthermore, it attains a 62.8%, 76.4%, 76.4%, 68.5%, and 66.4% improvement in device density for designing NOT, AND, OR, NAND, and NOR gates.

The total and average energy dissipation witness improvements of 15% for the proposed RND inverter gate, 52% for the AND gate, 59% for the OR gate, 30% for the NAND gate, and 18% for the NOR gate. Power and polarization analyses in QCAPro reveal that energy dissipation is lower in the proposed RND inverter than in existing inverters, marking it as one of the best and most innovative approaches for building and designing various digital circuits in QCA.

Furthermore, as manufacturing smaller QCA-based systems makes them more susceptible to faults and defects, the precise placement of individual cells with proper displacement in our proposed designs necessitates utmost care during fabrication. These issues introduce new research challenges, providing researchers with further opportunities to enhance high device density operational speed and reduce power dissipation.

### Ethics statement

Research does not include studies on human subjects, human data or tissue, or animals.

## Data Availability

All data generated or analyzed during this study are included in this published article.
